# Commercial hatchery processing may affect susceptibility to stress in laying hens

**DOI:** 10.1371/journal.pone.0291324

**Published:** 2023-09-11

**Authors:** Enya Van Poucke, Hedvika Suchánková, Per Jensen

**Affiliations:** IFM Biology, AVIAN Behavioural Physiology and Genomics Group, Linköping University, Linköping, Sweden; Ain Shams University Faculty of Agriculture, EGYPT

## Abstract

Directly upon hatching, laying hen chicks are exposed to multiple stressful events during large-scale hatchery processing, which may affect their later coping abilities. Commercial hatchery chicks (HC) were compared to chicks that were incubated and hatched simultaneously under calm conditions (CC). After being raised under similar, non-stressful conditions for 36 days, all chicks were exposed to a series of stressors: transportation and introduction into a novel environment followed by a regrouping event in order to characterize long-lasting consequences of hatchery treatment. Tonic immobility, corticosterone levels, and peripheral body temperature were used to assess reactions to the stress events. Tonic immobility was not affected by treatment but was significantly reduced in CC after transport. Corticosterone levels did not differ between treatments when assessed two days before and two days after regrouping. Comb temperature was significantly higher in HC following regrouping, indicating stress-induced hyperthermia. Furthermore, comb temperature dropped more following blood sampling in HC than in CC, indicating a stronger autonomic response to acute stress. In conclusion, the results suggest possible long-term negative effects of commercial hatchery processing, compared to hatching under silent and less stressful conditions, on the coping ability of laying hens to later stressful experiences.

## Introduction

In egg production, as in many animal farming systems, animals are often exposed to various degrees of potentially stressful events. Stress encountered early in life may have long-lasting consequences for the later behaviour and welfare of humans as well as animals [1]. For example, rats exposed to early stress show lasting adverse effects on neuroendocrine development, which affects their later cognitive and emotional development [2] and chickens with a history of stress during their first weeks of life are more anxious in different behavioural tests later in life [3, 4].

Laying hens typically experience a range of stressful events during their first day of life, related to standard commercial hatchery procedures [5], which may have important implications for their later welfare. Following incubation in large and noisy incubators (up to 90 dB, according to personal communication with commercial hatchery personnel), they are transported on conveyor belts to stations for sex sorting and vaccination before continued conveying, including transient speed increases, until loaded into crates for transport to rearing farms. Although previous studies on broiler chicks have failed to find significant effects on short-term welfare-related traits following conveyor handling [6], it has been reported that directly after hatching and later during the processing procedure commercially hatched chicks show increased corticosterone levels compared to chicks hatched under non-commercial (calm) conditions [5], and that the early stress exposure causes long-time effects on stress susceptibility and the emotional state of chickens [5, 7]. Hence, the treatment of chicks in large-scale hatcheries may affect their ability to cope with challenges later in life.

Under commercial rearing conditions, a range of new potentially stressful events emerge as the chicks leave the hatchery. First, they are transported for several hours loaded in crowded transport crates, then they are introduced to a novel environment on the rearing farm, which is combined with social regrouping where hundreds of new individuals are encountered. After rearing, they are faced with another road transport, this time to the production farm, and again this is followed by introduction to a novel environment combined with social regrouping. Although there is a lack of systematic data relating to the effects of these different practices, each of them can be considered a possible welfare risk, placing challenges to the birds’ ability to cope [8].

The importance of early experiences for later welfare has been known for a long time. To mitigate negative consequences, various attempts have been made to improve the long-term welfare of laying hens, for example, by enriching the rearing environment in order to stimulate a wider range of natural behaviour [4, 9, 10]. Some of the experiments do indeed reveal a positive effect, and there are indications that early enrichment may also buffer some of the stress from commercial hatching by making chickens more resilient against stress later in life [11], although the results are somewhat ambiguous.

Since very little is known about how the life-long welfare of billions of laying hens worldwide are affected by early stress, it is necessary to investigate how chickens that have been exposed to commercial hatching react to later-life stressors. To do so, we studied their responses to transport, the introduction to novel environments, and social regrouping and compared them to control chicks hatched under calm and less stressful condition. We used three different measures of stress responses: tonic immobility, corticosterone levels, and peripheral temperature.

Tonic immobility is a widely used measure of fear in chickens, which can give some useful indications on stress coping ability [12], while corticosterone gives an indication of the activity of the hypothalamic-pituitary-adrenal axis [13]. To obtain a measure of the other main aspect of the stress response, the sympathetic nervous activity, we also included non-invasive infrared thermography measurements of peripheral body temperature. Heavily vascularized peripheral body parts such as combs, eyes, and side of the face have been shown to be affected during acute stress in chickens [14, 21], and the effects can be moderated by early experiences [15].

The aim of this experiment was to assess the effects of early stress encountered during commercial hatchery routines on later stressful events that represent the types of challenges laying hens typically experience during rearing.

## Materials and methods

### Ethical note

All experimental protocols were approved by Linköping Council for Ethical Licensing of Animal Experiments, ethical permit No. 14916–2018 (Linköping, Sweden). Experiments were conducted in accordance with the ARRIVE guidelines. The protocol was performed in accordance with the relevant guidelines and regulations.

### Animals, hatching, and housing

All chickens included in this study were female White Leghorns from the Lohmann LSL strain (Lohmann Tierzucht GmbH, Germany). Both experimental and control chickens originated from the same parental flock and hatched from eggs that were collected and incubated at the same time point. 105 female chicks (hatchery chicks, HC) were hatched at the commercial hatchery in southern Sweden before being transported for 4 h by truck to Linköping University without the provisioning of water. The large-scale incubator at the commercial hatchery contained about 60,000 eggs and uses fans generating up to 90 dB of noise in order to circulate air to maintain a uniform temperature throughout the space. Standardized hatching and handling routines at commercial hatcheries include sex sorting, vaccination, conveying, and loading for transport as previously described by [5]. Upon arrival at the university the chicks were weighed, leg-ringed, and placed in a pen with sawdust, a heat lamp, and water. On day 9 post-hatch HC were wing-tagged and sham-vaccinated to control for the vaccination handling control chicks experienced.

Before the onset of incubation 270 fertilized eggs were collected from the same batch and parental stock as HC and transported to Linköping University to serve as control chicks (CC) that were hatched under non-commercial (calm) conditions. These eggs were placed in an incubator at the exact same time as HC eggs. The incubator (Masalles Incubator Mod.25-I HLC) can hatch 200 eggs and is nearly silent due to the small fan needed to maintain 37.8°C and 55% humidity. A technical problem with one of our incubators led us to discard about 100 eggs. After hatching, the chicks were removed from the incubator at a corresponding time as HC and wing-sexed. All males were discarded, so we ended up with 71 female control chicks that were placed in a pen with sawdust, a heat lamp, and water until the arrival of HC. CC were weighed and leg-ringed at the same time as HC. On day 9 post-hatch CC chicks were wing-tagged and vaccinated against Marek’s disease (HC were sham-vaccinated since they had already been vaccinated at the hatchery). Additionally, on day 13 and 28 post-hatch CC chicks were vaccinated against coccidiosis and infectious bronchitis serotype Massachusetts respectively, by adding vaccine to the drinking water preceded by two hours of water deprivation.

Without mixing treatment groups, a total of 105 HC and 71 CC were distributed into four rearing pens of 90x180 cm after hatch (HC1, *n* = 53; HC2_,_ *n* = 52; CC1, *n* = 36; CC2, n = 35). Each pen contained wood shavings, a heat lamp, and *ad libitum* feed and water. On day 9 post-hatch, perches were added to each of the rearing pens, and on day 23 all pens were expanded to 90x270 cm. The light regime was kept on a 12:12 h light-dark cycle.

### Experimental procedure

The experimental treatments to be investigated started after the initial 35 days of common rearing of both HC and CC, and continued until the birds were 67 days old. During this period, the three treatments were successively applied to all birds at the same time: transport, novel environment and social regrouping, as detailed below. After each of the treatments, measures reflecting the coping ability of the birds were taken, as outlined under “Measurements” below.

On day 36 post-hatch both treatment groups were transported from our hatching facility to a larger research facility. This is normal breeding routines in our facilities, but at this occasion the transport was extended in order to closer resemble the type of transport that commercial chickens are exposed to. Hence, the transport was considered to be a stressor.

Chicks within each rearing pen were randomly divided into two large cardboard boxes (58x35x41 cm) with ventilation holes. A total of eight boxes were distributed among two passenger cars with internal air conditioning set at 20°C. Each car transported two HC and two CC boxes. Both cars drove the same route at the same time, driving for a total of approximately one hour on both highways and smaller local roads.

Upon arrival, the birds were distributed into four equally sized pens, 120 x 360 cm, in the same groups as they were kept in before the transport. Each pen was covered with wood shavings and was equipped with water and feed *ad lib* as well as two perches. The light regime was 12:12 h light:dark. This procedure constituted an additional stressor, i.e., introduction to a novel environment.

On day 58 post-hatch, 22 days after arrival to the new facility, the chicks underwent a third potentially stressful procedure, i.e., regrouping to mix up the existing social groups. Each treatment group was housed in two pens. All chicks within a treatment group were hand-caught and put into holding crates before being redistributed into their new social groups. Half of the individuals from each original pen were allocated to the other pen of the same treatment group, consequently mixing the social groups but keeping the treatment groups intact. By means of this, we ended up with a total of two pens per treatment with new social composition. The experiment ended when the chickens were 67 days old.

### Measurements

#### Weight

Chicks were weighed at hatch (*n* = 176), and at 8 (*n* = 173), 15 (*n* = 173), 22 (*n* = 173), 29 (*n* = 173), 36 (*n* = 173), 43 (*n* = 173), 50 (*n* = 173), and 56 (*n* = 173) days of age. Weighing was done on a scale with a precision of 0.01 g (Denver Instruments MXX-612) for the first 30 days, and thereafter with a precision of 0.1 g (Radwag WLC 6/C1/R).

#### Tonic immobility

Tonic immobility (TI) tests were performed on days 35 and 37, i.e., the day before and after the transportation on day 36. Twenty chicks from each treatment were randomly selected (10 from each pen) and the same birds were tested on both occasions. The testing was done in a separate room where the birds had no visual or acoustic contact with other birds. All testing was done blindly, where one person collected a chick, and another performed the actual test without knowing which treatment group the bird came from. The test was performed using the method outlined in detail previously [7]. Briefly, each chick was placed on its back in a wooden cradle while holding them with one hand lightly on their thoracic-abdominal region and applying a slight pressure for 10 s. If the chick stood up within five seconds, they were not deemed to have entered TI, and the induction attempt was repeated a maximum of two more times. All tests were video recorded, and afterwards, we recorded the time until righting. The maximum time until righting was set to 600 s.

#### Behavioural assessment at regrouping

In connection with regrouping (which was performed on day 58) observations of the behaviour of each group were performed. Since there were only two pens per treatment, it was not possible to carry out proper statistical testing of the results, so the observations primarily had the purpose to document that the regrouping actually caused the intended increase in aggression and stress related behaviour. The observations were done on days 53, to obtain a baseline, on day 58, starting one hour after regrouping, and on day 62, to assess any long-time effects of the treatment. At all three occasions, observations were carried out during 60 min between 13.00 and 15.00 h. Two observers alternated between the four pens in a balanced manner and used 1/0-sampling with 30 s intervals to record the occurrence of the predefined behaviours on a group level. This means that during each 30 s interval it was recorded whether the defined behaviour was observed in the group or not. Observers synchronized on all behaviours in the ethogram during a pilot behavioural observation session. The recorded behaviours, as well as the results from the observations, are listed in S1 and S2 Tables.

#### Corticosterone measurements

The effect of regrouping on the activity of the hypothalamic-pituitary-adrenal (HPA) axis was determined by obtaining blood samples for corticosterone (CORT) assessment in the morning on day 56 (two days before regrouping, baseline CORT) and again on day 60 (two days after regrouping). A total of 22 birds from each treatment group (HC and CC) were randomly selected from their pens for blood sampling and the same birds were sampled on both occasions. The birds were collected from their pens while the light was out (birds were then picked in darkness at random) and carried to another room where they were blood sampled by venipuncture of the brachial vein using hypodermic needles (B. Braun Sterican 23G 0,60x30mm) within 3 minutes of gently hand-catching in darkness. The blood was collected using Microvette heparinized 200 μl tubes. Blood samples were immediately centrifuged, the plasma was separated and then stored at -20°C until further analysis. The CORT analysis was performed with ELISA, using kits from Enzo Life Sciences. The samples were measured in duplicate and analyzed according to the product manual: http://static.enzolifescience.com/fileadmin/files/manual/ADI- 900–097_insert.pdf.

#### Measurement of peripheral body temperature

To assess the autonomic stress reaction related to the regrouping procedure, we measured peripheral body temperature before and after regrouping on three different time points as outlined below.

Thermal imaging using an infrared camera (FLIR T540) was performed in conjunction with blood sampling (see above). All individuals subjected to blood sampling were also used for thermal imaging (22 birds from each treatment). On day 56 post-hatch, directly after being blood sampled, individuals were gently kept in an upright position on a table. For each chick, multiple thermal images of the side of the head were promptly taken from 1 m away. On day 60 post-hatch, two days after regrouping, the same individuals were thermally imaged again with the same method. This time imaging took place both before and after blood sampling. Hence, we obtained images at three different time points: baseline (before regrouping), after regrouping (before blood sampling) and after regrouping (after blood sampling as an additional stressor).

**Fig 1:**
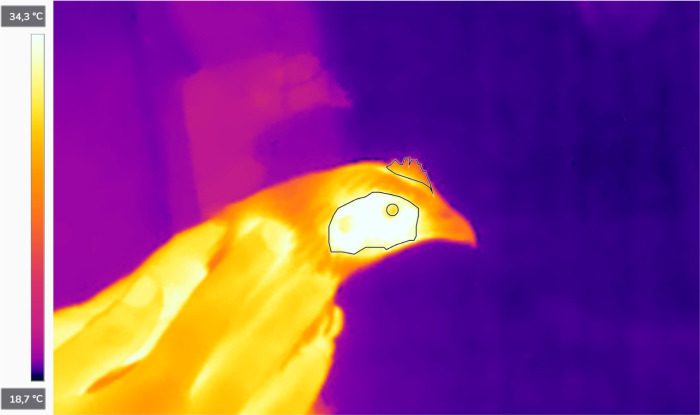
Infrared image showing the outlined comb, eye, and cheek area used for thermal measurements.

All thermal imaging was completed within 2 minutes of blood sampling and 4 minutes of gently hand-catching in the pen. Emissivity was set to 0.98, which is FLIR T540’s recommended standard setting for human skin. Ambient room temperature and relative humidity levels were recorded (Rubicson kompact digital hygrometer) prior to imaging each individual and adjusted accordingly in the camera settings. Image analysis was completed using FLIR Thermal Studio software. Three heavily vascularized areas for thermal measurement were manually outlined per image: comb, eye, and cheek (defined as the feather-less “ear-lobe” area on the side of the head) (Fig 1). A maximum, minimum, and average temperature was obtained for each of the abovementioned areas, and the average temperature was then used for further analysis. For every imaging instance, two sharp images per individual were analyzed and their values averaged.

### Statistics

Generalized Linear Models (SPSS 29.0) with linear scale response and link function “normal” were used after checking the normality of the distributions using visual inspections of P-P and Q-Q plots. The models included hatchery treatment (HC vs CC) and age where relevant, and when measures were repeated on the same individuals (tonic immobility, corticosterone levels, peripheral temperatures), a repeated measures design was used including time-point as a model variable (Generalized Linear Mixed Models, SPSS 29.0). For direct comparisons between treatment groups (for the variable weight), and post hoc tests, t-test was used. Pearson correlations were calculated for the associations between temperatures measured on different body parts. The behavioural data were collected as a qualitative assessment of the effects of regrouping, and were not subjected to any statistical evaluation since there were only two groups per treatment. The complete data set can be found in S2 Table.

## Results

### Weight

CC were significantly heavier than HC on day 1 (mean ± SEM: 35.3 ±0.22 vs 37.6 ±0.28 g; *t*_174_ = -5.881, *p* < 0.001). There were no significant differences between the average weights of CC and HC on any other of the weighing days, and no significant interactions between treatment and age.

### Tonic immobility: Transport effects

**Fig 2:**
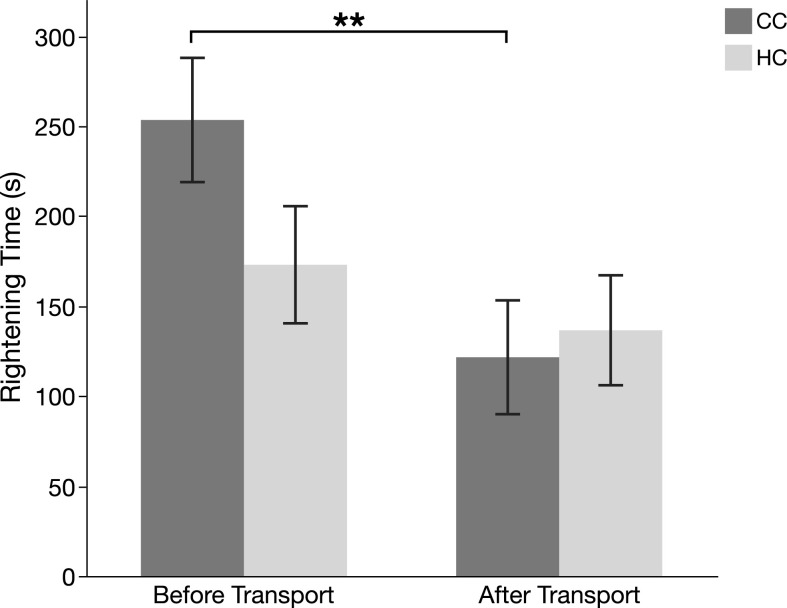
Mean rightening time (± SEM) during tonic immobility in hatchery chicks (HC) and control chicks (CC). ** P ≤ 0.01.

There was no effect of treatment (HC vs CC) on righting time (Fig 2; Treatment effect: *F*_1,76_ = 1.02, *p* = 0.315), and there was no significant interaction between treatment and transport (*F*_1,76_ = 2.18, p = 0.14). However, righting times were significantly shorter after transport (*F*_1,76_ = 6.77, *p* = 0.011). From Fig 2, it can be seen that this was mainly explained by a reduction in righting time in CC, while there was no significant difference in righting time before and after transport in HC (post-hoc test of difference between before and after transport for CC: *t*_38_ = 2.82, *p* = 0.008; HC: *t*_38_ = 0.82, *p* = 0.42).

### Behaviour at regrouping

Since there were only two groups per treatment, and the behavioural observations were carried out on a group level, no statistical analysis of the results are possible. Hence, the numbers should be taken only as an indication whether the animals were in fact affected by the regrouping in the way that was expected. The results are summarized in S1 Table.

Overt aggression was observed more often in both groups at the day of regrouping, whereas this had gone back to baseline numbers one week later. Sparring was reduced during regrouping but had also returned to baseline one week later. Severe feather pecking was markedly increased in both groups, particularly in HC, during regrouping, and remained at a higher level than baseline one week later, whereas gentle feather pecking was reduced during regrouping and remained at a lower level than baseline one week later.

#### Corticosterone assessment

**Fig 3:**
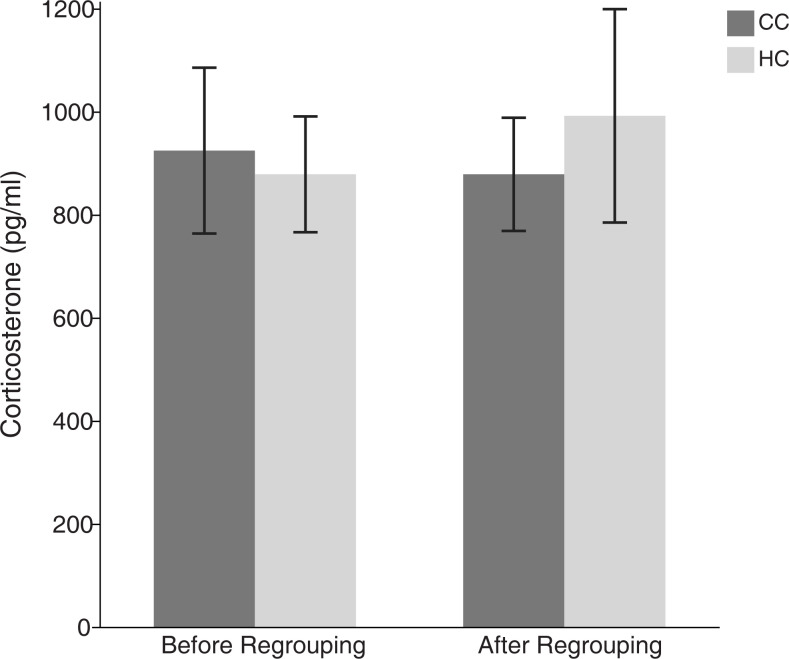
Mean levels of corticosterone (± SEM) before and after regrouping in hatchery chickens (HC) and control chickens (CC).

There were no significant differences between HC and CC in the CORT values, and no differences between the values before and after the regrouping event (Fig 3; treatment effects: *F*_1,77_ = 0.005, *p* = 0.94; effects of regrouping: *F*_1,77_ = 0.14, *p* = 0.71). There was also no significant interaction (*F*_1,77_ = 0.28, *p* = 0.60).

#### Peripheral body temperature

**Fig 4:**
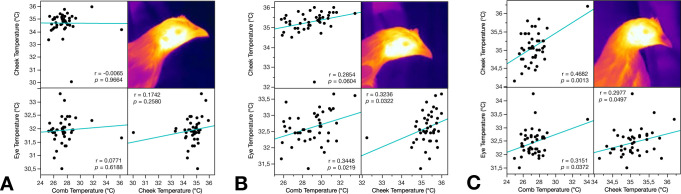
Correlation plots of comb, cheek, and eye temperatures in all individuals **A** two days before regrouping; **B** two days after regrouping, immediately before stressor; **C** two days after regrouping, immediately after stressor.

The temperature measures of different peripheral body parts (comb, cheek, eye) were mostly correlated when measured at the same occasion on the same individual. The correlations are depicted in Fig 4 as indications of the validity of the measurements.

**Fig 5:**
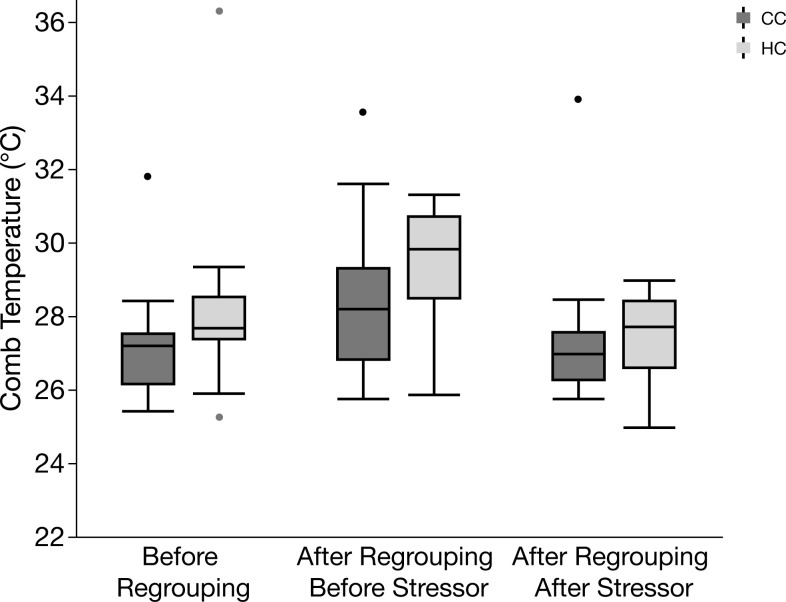
Box plot of comb temperature before regrouping, after regrouping before stressor, and after regrouping after stressor in hatchery chickens (HC) and control chickens (CC). Median values with 25^th^ percentiles indicated by the boxes; whiskers show max and min values, and occasional outliers are indicated by dots.

**Fig 6:**
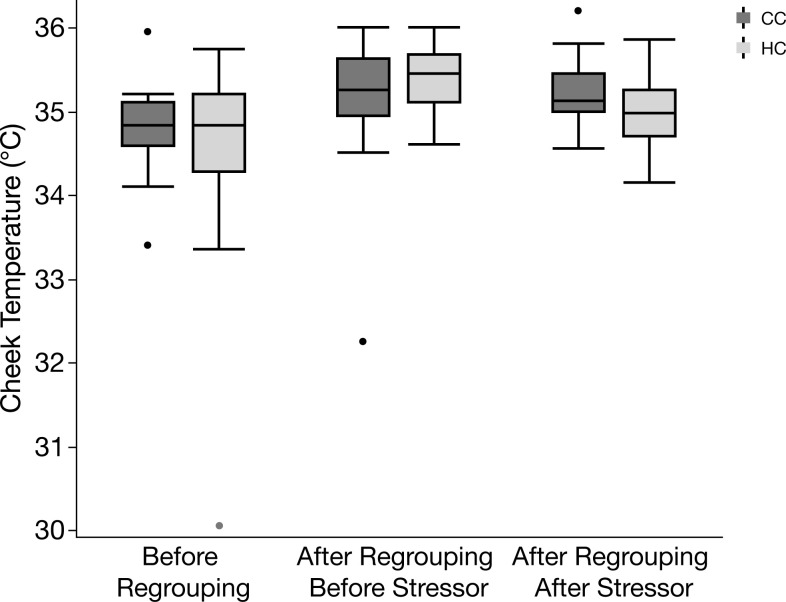
Box plot of cheek temperature before regrouping, after regrouping before stressor, and after regrouping after stressor in hatchery chickens (HC) and control chickens (CC). Median values with 25^th^ percentiles indicated by the boxes; whiskers show max and min values, and occasional outliers are indicated by dots.

**Fig 7:**
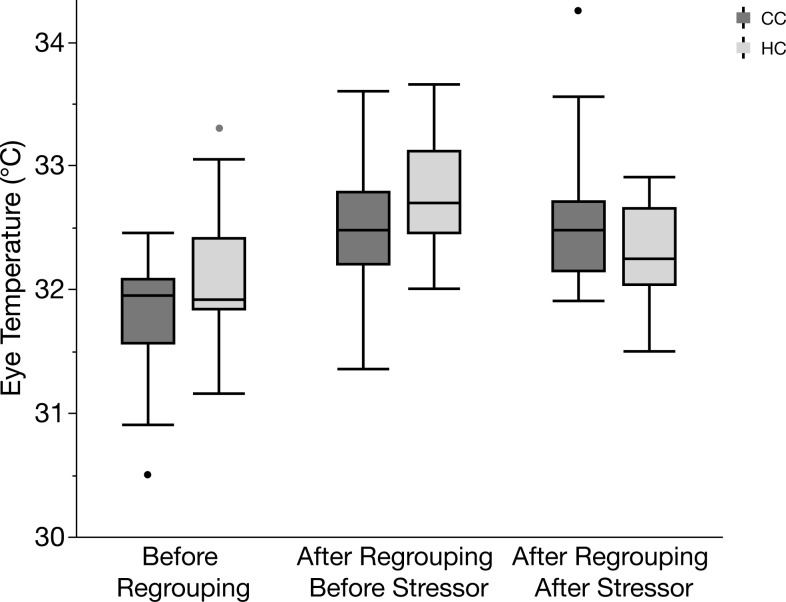
Box plot of eye temperature before regrouping, after regrouping before stressor, and after regrouping after stressor in hatchery chickens (HC) and control chickens (CC). Median values with 25^th^ percentiles indicated by the boxes; whiskers show max and min values, and occasional outliers are indicated by dots.

The effects of treatment and regrouping on the peripheral body temperatures are shown in Figs 5–7. There was a significant effect of time point and treatment on comb temperature (Fig 5), being higher after regrouping and blood sampling and also significantly higher in HC after regrouping, while decreasing after blood sampling (effects of time point: *F*_2,126_ = 11.1, *p* <0.001; effects of treatment: *F*_1,126_ = 5.4, *p* = 0.013). Cheek temperature was also significantly affected by time point in the same way as comb temperature (*F*_2,126_ = 7.22, *p* = 0.001) whereas it was not affected by treatment (*F*_1,126_ = 0.7, *p* = 0.4) (Fig 6). Also, eye temperature was similarly affected by time point (*F*_2,126_ = 24.2, *p* < 0.001) (Fig 7) but again there was no overall effect of treatment (*F*_1,126_ = 1.3, *p* = 0.26). However, for eye temperature, there was a significant interaction between treatment and time point (*F*_1,126_ = 4.1, *p* = 0.02), caused by a stronger decrease after blood sampling in HC.

## Discussion

We determined how the stressful routines associated with commercial, large-scale hatching of laying hen chicks affected their susceptibility to various stressors occurring later in life. We found that the tonic immobility reaction was reduced following transport, mainly due to an effect observed in control chicks (CC) and not in hatchery chicks (HC). Furthermore, there was a significantly increased peripheral body temperature following regrouping in HC compared to CC, and a significant drop in comb temperature in HC after an acute stressor (blood sampling). However, we did not detect any effects of hatching conditions on corticosterone levels or weight gain. The results indicate that the previously demonstrated stress experienced during the first day of life in commercially hatched chickens may possibly have some long-lasting effects on the ability to cope with the types of challenges typically encountered by laying hens during a production cycle.

Commercial hatching of laying hen chicks is associated with several stressful events, such as hatching in large, noisy incubators, conveyor handling during sex sorting and vaccination, and long road transports to the rearing farms. Previously, we have found that this procedure is associated with acute stress reactions that have long-time effects on stress sensitivity and emotional states of laying hens [5, 7, 16, 17]. However, the challenges for chickens in egg production do not end there. After a growth period on the rearing farm, they are normally transported once again on lorries to production farms, where they encounter a new environment and are mixed with unfamiliar birds [18]. Each of these events present challenges to the coping ability of the birds, and this potentially affects their life-time welfare [8]. In the present study, we aimed to investigate whether the early stress associated with commercial hatching modifies the ability of chickens to cope with stressful events later in life. We deliberately chose stressors similar to those encountered later in life during typical chicken rearing, although we did not attempt to mimic the standard rearing procedures.

Under commercial egg production conditions, laying hens get transferred from the rearing to the laying facility at around 17 weeks of age [18]. In practice, this transport is immediately followed by a mixing of social groups. In order to dissect out the effects of these two procedures, we chose to separate them out in time. In accordance with our husbandry guidelines, chicks are transported from our rearing facility to our larger research facility at about 5 weeks of age, hence we chose to use that transport opportunity to see how transportation and introduction to a novel environment affected our two treatment groups. While our transport and regrouping procedures were not conducted at similar timepoints as they occur in the industry, valuable lessons can be gleaned about how exposure to commercial hatchery stress might affect how laying hens will react to production procedures they are exposed to later in life.

Here, we decided to model the different events in order to maintain a strict control over how the birds were treated. This meant that we most likely underestimated the magnitude of the stress associated with each event. The transport was conducted with the chicks in small groups and using passenger cars. Additionally, the novel environment where the chicks were introduced to after the transport was relatively non-challenging compared to large-scale commercial production units that usually contains many thousand birds in a limited space. Furthermore, the regrouping treatment was conducted in a way that only half of the birds in the new groups were strangers to the others, and the birds had plenty of space to escape and avoid conflicts compared to the situation in a commercial setting. Hence, the stress treatments were considered valid models of the practical rearing routines, but due to the experimental constraints were probably less challenging to the birds [18].

In the present study, the hatchery processed chicks (HC) weighed significantly less after transport from the hatchery to the rearing facility compared to control chicks (CC) which were hatched at the same time, something that we have observed in previous studies as well [11]. This could be due to water loss during transport, since CC were provided with water when they were removed from the incubators whilst HC spent several hours without water during the transport from the hatchery. However, it can also be a result of the stress of transport in the day-old birds. Whereas CC were significantly heavier on day 1, HC caught up in weight within a week and there were no obvious effects of the later transport and regrouping on growth.

Most research on transport stress in chickens has focused on broilers and the effects on meat quality parameters. However, it is clear that all transport of all sorts of poultry is associated with a range of potential stressors, such as handling, feed and water withdrawal, noise, vibrations, and crowding [19]. So it is clear that stress reactions to transport do occur in chickens [19], but our present results indicate that commercial hatchery processing did not significantly affect this. Possibly, the relatively short transport used in this experiment was not severe enough to affect weight development.

We used tonic immobility (TI), a well-established behavioural test of fearfulness and anxiety in chickens, to further elucidate reactions to transport. The test was conducted one day before and one day after the transport to the larger facility with the assumption that TI would increase after transport. However, we found the opposite, TI righting times were in fact significantly shorter following transport, mainly caused by a large and significant reduction in CC. One possible interpretation of our results is that the testing conditions in the larger facility were different from the ones where the birds were kept before transport, which may have reduced the overall TI response. It is also possible that the birds were habituated to the TI procedure, since they had already experienced the same test once before. The fact that CC birds, but not HC, had a significant decrease in TI after transport may therefore indicate that CC were less negatively affected by the transport.

Whereas TI is an often used and validated measure of fear and anxiety that reliably reflect the stress that a bird has been exposed to prior to testing, it is known to be highly susceptible to various external factors [12]. For example, the light and sound conditions in the test room, as well as the way the birds are handled immediately before the test all affect the actual duration of TI.

We then used systematic regrouping as a further model of a typical stressful event under commercial conditions. Due to practical constraints with space and pens, we had to perform this regrouping so that we only used four pens in total, which meant that we had only two groups per treatment (HC and CC). Hence, it was not possible to perform any proper statistical analysis of the behavioural reactions to the regrouping, since each group by necessity must be regarded as the statistical replicate unit. The behavioural observations were conducted only to verify that regrouping caused the expected increase in aggressive behaviour, indicative of the stressfulness of the procedure [20]. As could be expected, we noted that aggressive behaviour and feather pecking increased after regrouping, indicating that the procedure was indeed perceived as challenging by the birds. Although not evaluated statistically, these effects were numerically stronger in HC.

We did not observe any effects of the regrouping on the levels of corticosterone, something that might have been expected based on previous research [20]. It is possible that the regrouping procedure we used was not as severe as under commercial conditions, since the birds had more space and possibilities to escape and avoid agonistic encounters than they would have had under commercial housing conditions. Previously, we have found that the reactivity of the HPA-axis is higher in hatchery processed chickens [5], but in the present study we only measured the baseline values two days before and after regrouping. It is possible that the HPA activity, if it was in fact reacting acutely to the regrouping, had returned to baseline on the second measurement.

We also measured the autonomic response to regrouping by assessing peripheral body temperatures with infrared thermography. These measurements were mostly correlated across the different body parts, indicating that the method we used was a reliable indicator of the autonomic response. HC were more affected by the regrouping and the blood sampling procedures, as shown mainly by comb and eye temperatures, again indicating a higher stress susceptibility. The typical response to an acute stress event in chickens is an initial, transient reduction in peripheral temperatures as blood is redirected to the core of the body, followed by an increase as a result of a general and sustained rise in body temperature (known as stress-induced hyperthermia) [21].

The relationship between early experiences and later effects on stress-induced hyperthermia has been demonstrated in previous studies, showing that chickens raised in enriched conditions have a less acute temperature response than chickens from a barren environment [15]. This is consistent with the findings in our experiment, where HC had a higher basal comb temperature two days after regrouping, indicating stress-induced hyperthermia, and a steeper reduction in comb temperature following the acute stress event of blood sampling, indicating a stronger autonomic response to stress.

Together, our results indicate that the stress associated with processing in a commercial hatchery may have made chickens more sensitive to stressful events occurring later in life. We have previously found that hatchery processing causes increased sensitivity of the HPA-axis and induces a “pessimistic” emotional state indicating poorer welfare [5, 17]. The present results indicate that commercially hatched chicks may possibly be more susceptible in terms of fearfulness and stress to the kinds of challenges that they are bound to encounter during practical egg production. However, this study simply served as a model and additional research exactly replicating poultry production procedures needs to be conducted in order to confirm these findings. It should also be noted that early stress might possibly in some cases prime chickens for coping with stress later in life, something that should be examined in future research in the field.

Some caution in the interpretations should be noted. Due to a technical error with one of the incubators, we ended up with fewer chicks in the control group. This resulted in a difference in group size and—subsequently—stocking densities between the treatment groups. It needs to be noted that differences in stocking densities have previously been shown to alter anxious behavior, CORT levels, feather pecking, and feather damage [8].

## Conclusion

Chickens processed at a commercial hatchery had a stronger autonomic response to regrouping and blood sampling. Tonic immobility responses indicated possible increased susceptibility to transport stress in chickens processed at a commercial hatchery. Commercial hatchery processing may therefore possibly have long-time negative effects on the ability of laying hens to cope with various stressful experiences associated with typical egg production procedures.

## Supporting information

S1 Table Behaviour at regrouping. Frequencies of behaviours exhibited in percent of scan observations before (day 53), at (day 58), and after (day 62) regrouping in hatchery chickens (HC) and control chickens (CC). Ethogram of behaviours.
(DOCX)

S2 Table The complete data set. (XLSX)
