# Supplementary material for: Commercial hatchery processing may affect susceptibility to stress in laying hens
Source: PLoS One. 2023 Sep 11;18(9):e0291324. doi: 10.1371/journal.pone.0291324 (PMC10495002; doi:10.1371/journal.pone.0291324)
Supplement: S1 Table — Frequencies of behaviours exhibited in percent of scan observations before (day 53), at (day 58), and after (day 62) regrouping in hatchery chickens (HC) and control chickens (CC). Ethogram of behaviours. (DOCX) [file pone.0291324.s001.docx]

**S1 Table. Ethogram for behaviours at regrouping.** Ethogram used during behavioural assessments at regrouping.

| **Behaviour** | **Description** |
| --- | --- |
| Aggression | Frontal displays with raised hackles towards the other birds head pecking, jumping or kick at other bird |
| Severe Peck | Hard and fast pecks and/or pulling at other birds’ feathers |
| Gentle Peck | Light, repeated pecks at the feathers of another bird |
| Spar | Frontal displays, often accompanied by little jumps |
